# Supplementary material for: A pH-gated conformational switch regulates the phosphatase activity of bifunctional HisKA-family histidine kinases
Source: Nat Commun. 2017 Dec 13;8:2104. doi: 10.1038/s41467-017-02310-9 (PMC5727384; doi:10.1038/s41467-017-02310-9)
Supplement: Supplementary file 1 — Supplementary Information [file 41467_2017_2310_MOESM1_ESM.pdf]

Supplementary Information

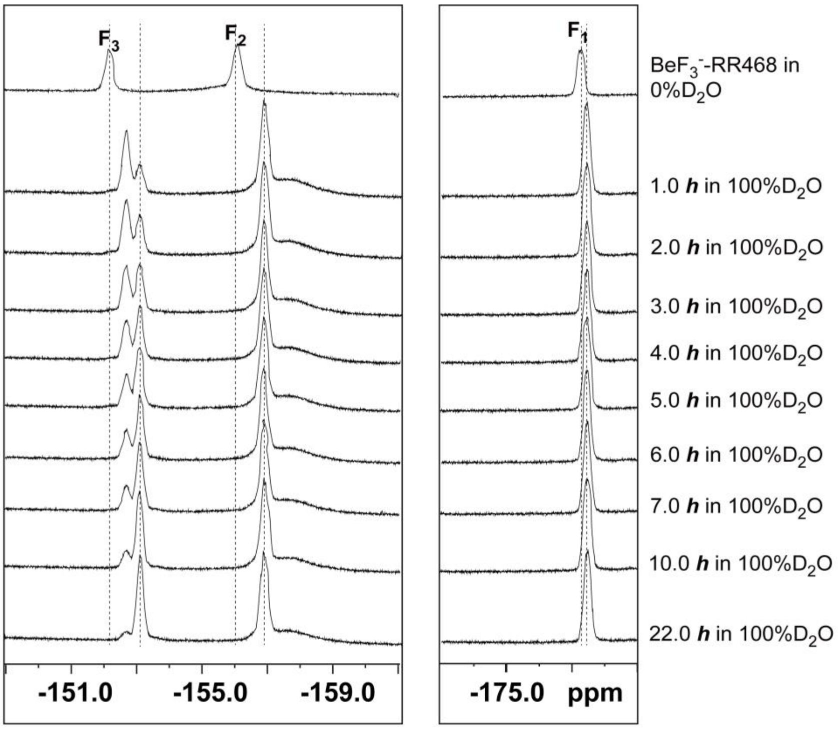

Supplementary Figure 1 | Hydrogen-deuterium exchange and solvent-induced isotropic shift experiments on BeF_3^--RR468. The ^{19}F NMR measurements were performed at 25℃ and pH 7.0.

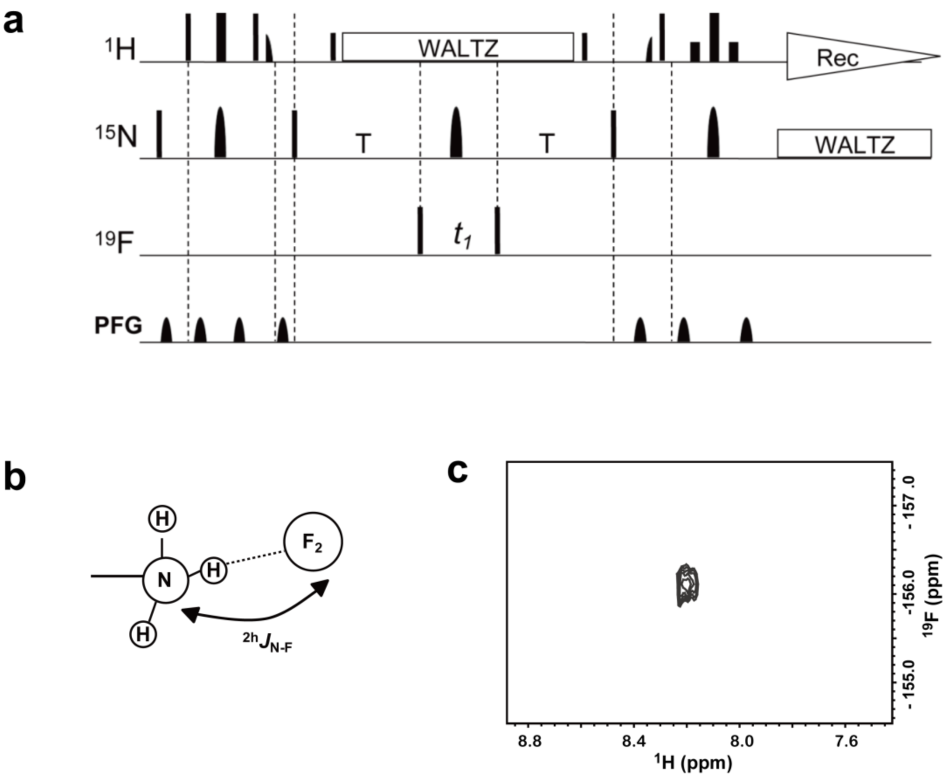

Supplementary Figure 2 | Visualization of the salt bridge between F_2 and the NH_3 side chain of K105 by the H3(N)F experiment. (**a**) The pulse sequence of the H3(N)F experiment. The experiment was adapted from the H3(N)P experiment[1] originally designed for analyzing the $^{3h}J_{NP}$ coupling by replacing the phosphorus channel with the fluorine channel. For the ^{19}F dimension, the carrier position was set to -156.0 p.p.m. The evolution delay of T in the ^{15}N dimension was set to 35.0 ms. (**b**) The schematic illustration of the weak two-bond *J* coupling of hydrogen bond. (**c**) The H3(N)F spectrum of the weak salt bridge between F_2 and the NH_3 side chain of K105.

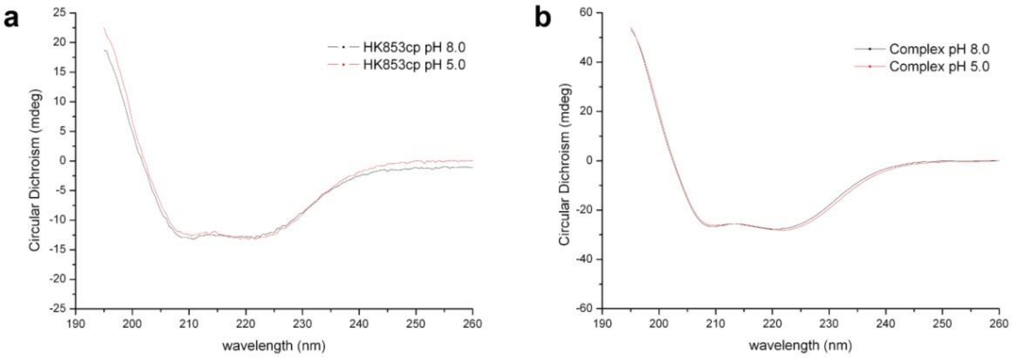

Supplementary Figure 3 | CD spectra of HK853^{cp} (a) and its complex with RR468 (b), at pH 8.0 and 5.0. The concentration of the HK853^{cp} is 0.3 mg ml^{-1} and the ratio of HK853^{cp} : RR468 is 1:1.5.

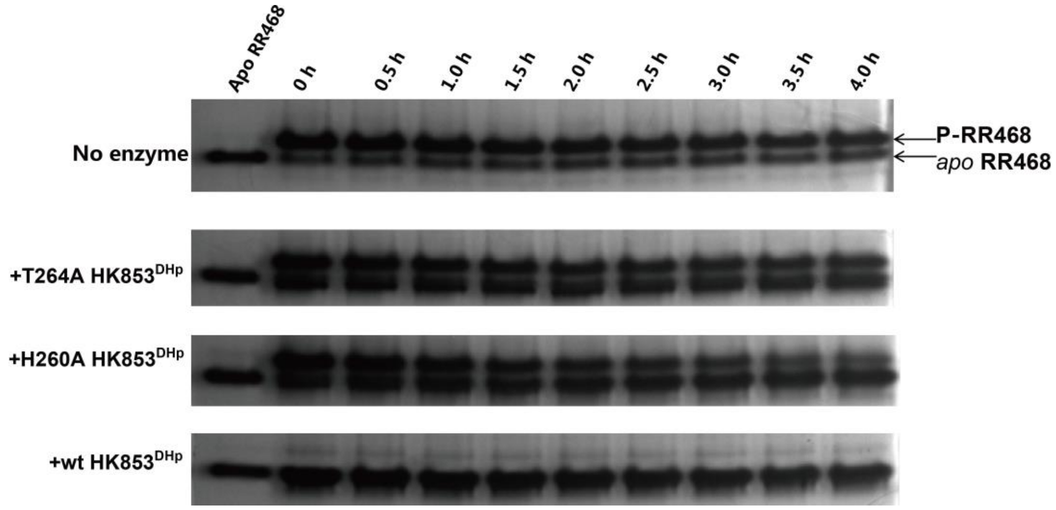

Supplementary Figure 4 | Phosphatase activity assays of wild-type HK853^{DHp} and mutants. The phosphatase activities were detected by the native-page gel at pH 8.0 at 4 ℃. The uncropped gel images are shown in **Supplementary Figure 5**.

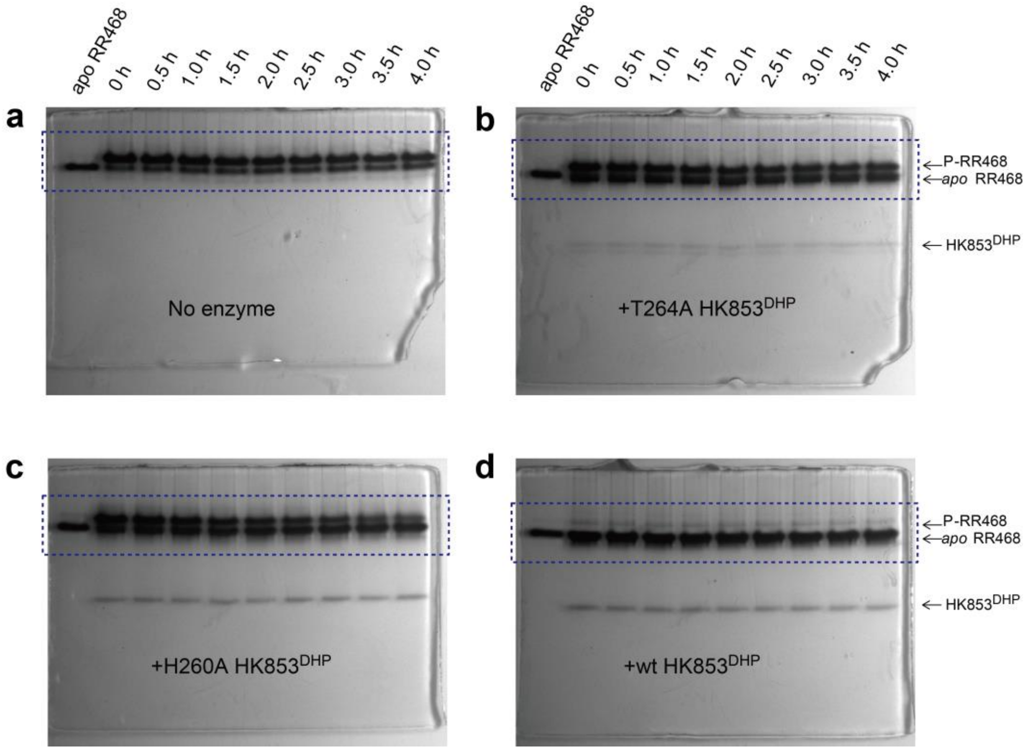

Supplementary Figure 5 | The uncropped images of the native gels of phosphatase activities of wild-type HK853^{DHp} and mutants. The assay without enzyme was conducted as a control shown in panel a. The phosphatase assays with enzymes were conducted with T264A HK853^{DHp} shown in panel b, H260A HK853^{DHp} in panel c, and wild-type HK853^{DHp} in panel d, respectively. The regions in the dashed boxes are retained in **Supplementary Figure 4**.

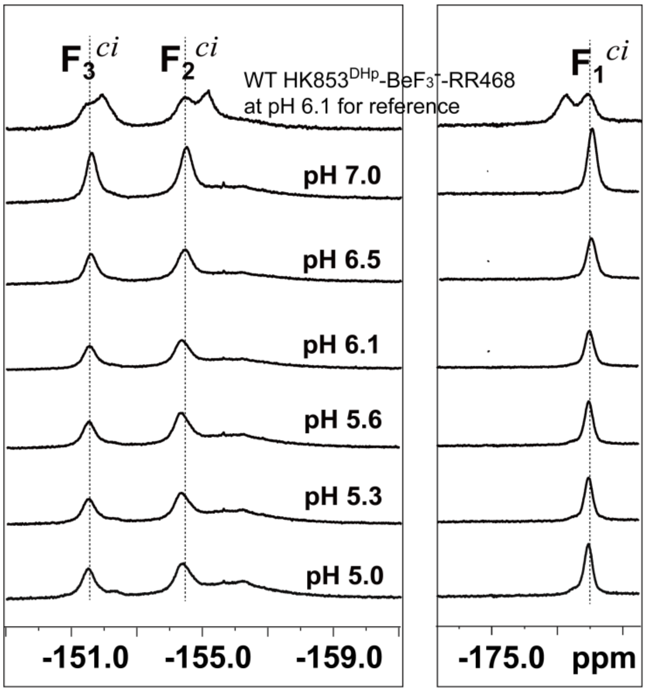

Supplementary Figure 6 | The ^{19}F NMR spectra of the H260A HK853^{DHp}-BeF$_3^-$-RR468 complex at different pHs. The spectrum of the wild-type HK853^{DHp}-BeF$_3^-$-RR468 complex at pH 6.1 (top row) was shown as a reference.

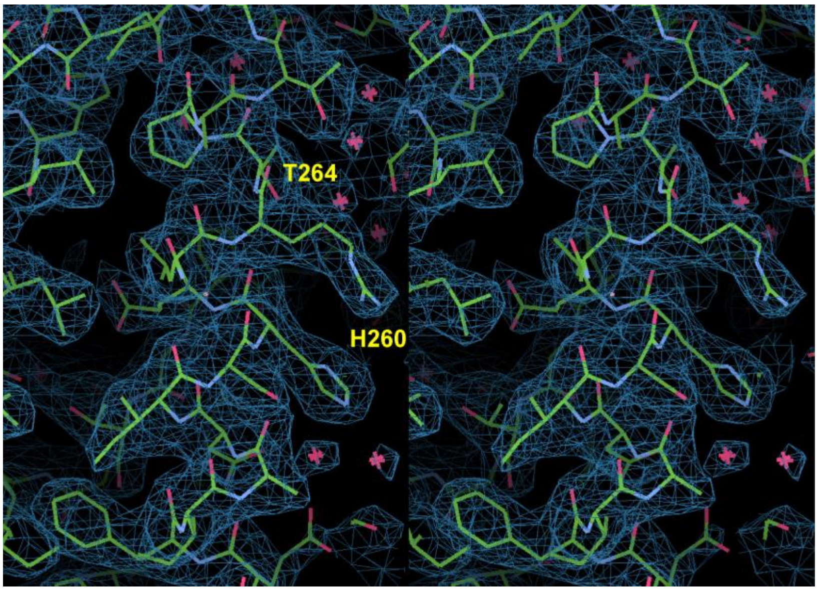

Supplementary Figure 7 | A stereo view of the 2Fo-Fc electron density map of the HK853^{cp}-BeF$_3^-$-RR468 complex reveals a catalytically inactive, *gauche*- χ^1 rotameric conformation of H260 at pH 5.0. The electron density map is contoured at 1.0 σ.

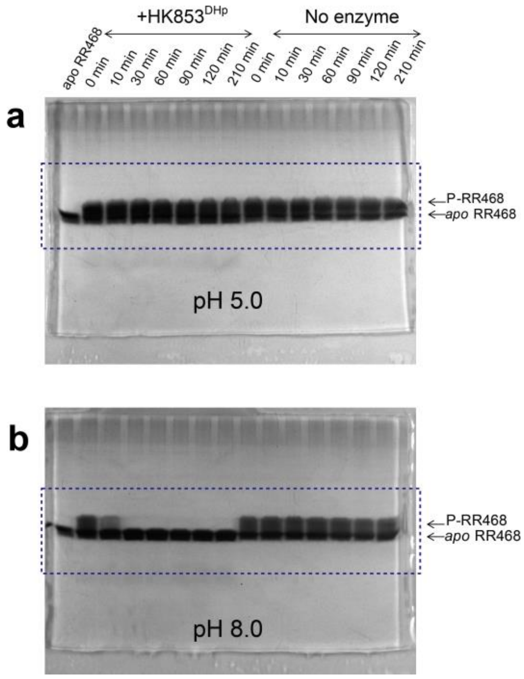

Supplementary Figure 8 | The uncropped gel images of HK853^{DHp} phosphatase activity at different pHs. The Phos-tag assays at pH 5.0 and pH 8.0 are shown in panel a and b, respectively. The regions in the dashed boxes are retained in **Figure 2e** and **2f**, respectively.

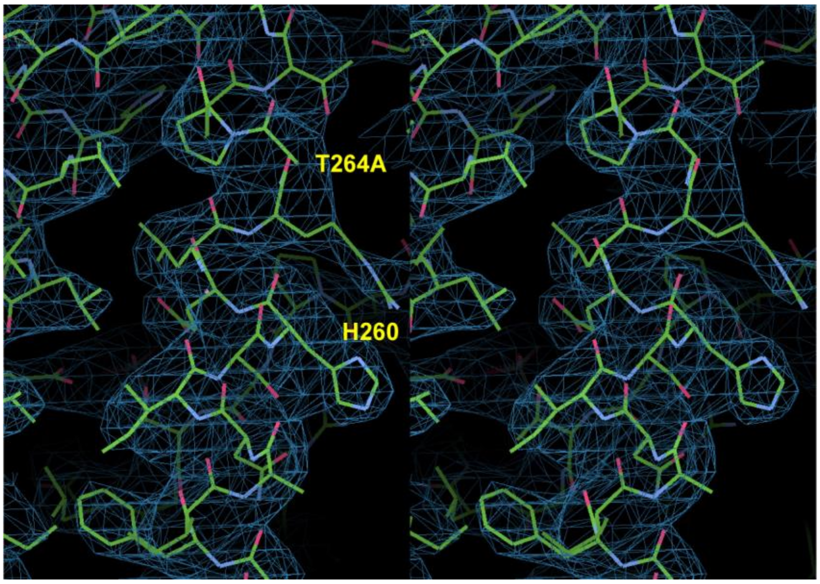

Supplementary Figure 9 | A stereo view of the 2Fo-Fc electron density map of the T264A HK853^{cp}-BeF$_3^-$-RR468 complex reveals a catalytically inactive, *gauche-* χ^1 rotameric conformation of H260 at pH 5.0. The electron density map is contoured at 1.0 σ.

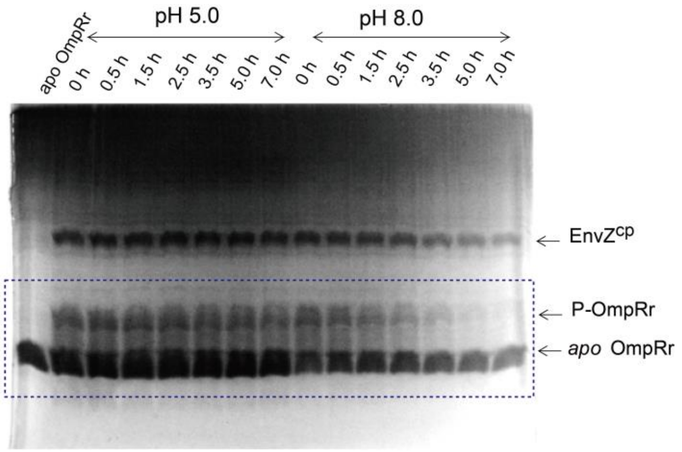

Supplementary Figure 10 | The uncropped gel image of EnvZ^{cp} phosphatase activity at pH 5.0 and 8.0. The region in the dashed box is retained in **Figure 4c.**

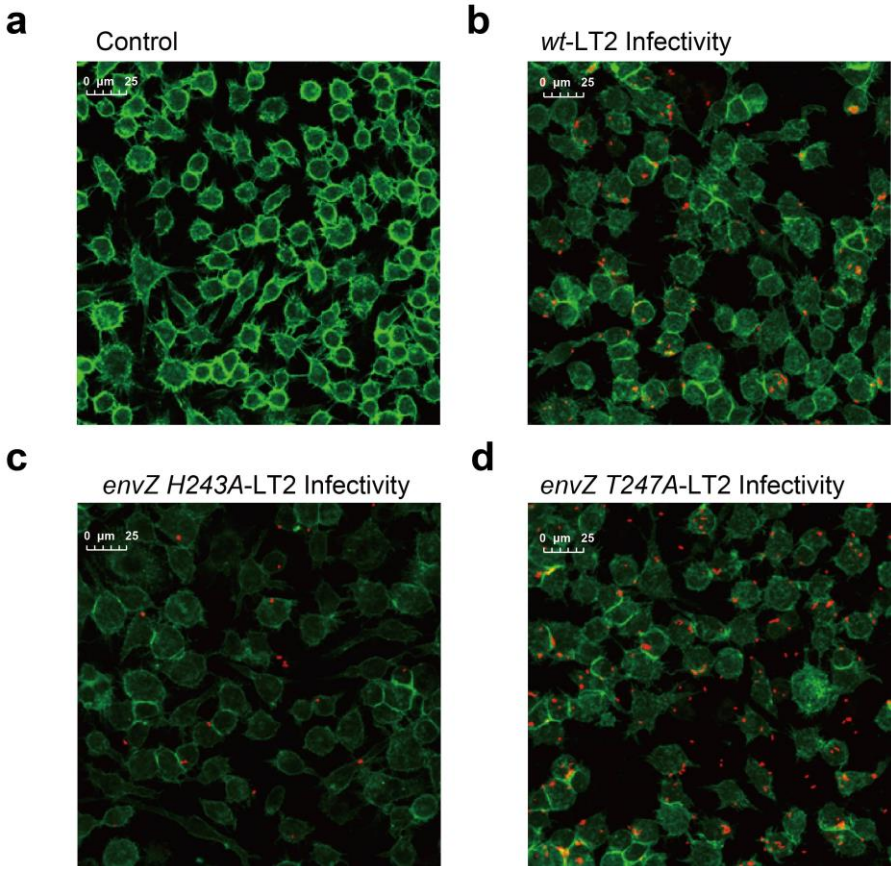

Supplementary Figure 11 | Representative images of macrophages infected by wild-type *S. typhimurium* LT2 and *envZ* mutants. (**a**) The RAW 264.7 macrophages cultured at 37 ℃. (**b**) The RAW 264.7 macrophages infected by wild-type *S. typhimurium* LT2. (**c**) The RAW 264.7 macrophages infected by the *envZ H243A S. typhimurium* LT2. (**d**) The RAW 264.7 macrophages infected by the *envZ T247A S. typhimurium* LT2. All images were visualized and processed by confocal microscopy. Macrophages are stained in green, and *S. typhimurium* LT2 cells are in orange.

Supplementary Table 1: Solvent induced isotopic shift (SIIS) of ^{19}F NMR for BeF_3^--RR468

F_x	F_1-(binding Mg^{2+})	F_2-(binding K105 and A84)	F_3-(binding T83 and M55)
SIIS ($\delta_{100\%}$ - $\delta_{0\%}$)	117.9 Hz	456.7 Hz	534.6 Hz

Supplementary Table 2: The primer sequences of HK853 mutations

Mutations	Sequences
H260A	Primer 1: CGAACATCTCGGCCGAGCTCAGAACGCCTTTAAC<br>Primer 2: CGTTCTGAGCTCGGCCGAGATGTTCGCTATGAAC
T264A	Primer 1:<br>GAACATCTCGCACGAGCTCAGAGCGCCTTTAACGGCCATAAAAGC<br>Primer 2:<br>GCTTTTATGGCCGTTAAAGGCGCTCTGAGCTCGTGCGAGATGTTC

Supplementary Methods

Procedures and primers for constructing *Salmonella envZ* mutant strains

Briefly, the DNA fragments carrying the mutated *envZ* gene (H243A or T247A) and its flanking DNA regions (about 500 bp) were obtained by overlapping PCR using the LT2 genomic DNA as the template. These two fragments were inserted into the suicide plasmid pDM4[2] by One Step Cloning Kit (Vazyme). The constructed plasmids were subsequently transformed into *E. coli* S17-1 *λ-pir*, which were used as the donor strains in conjugations with *S. typhimurium envZ::kan* strain. Colonies resistant to kanamycin and chloramphenicol were checked for the integration of each pDM4 derivate, and then spread onto the sucrose containing plate to select the second DNA cross-change. Colonies that lost all antibiotic resistances were selected for mutant confirmation by a combination of PCR and DNA sequencing analysis.

Primers used for constructing *Salmonella envZ* mutant strains are listed below:

envZ-Kan-F: GGCGTTTAACCATATGGCAGCCGGCGTGAAGCAATTGGCCGA GATTGCAGCATTACACG

envZ-Kan-R: ATTGATCGACTCCGCGAGATAACCGTCTTCCTCGCCCATCATG TAACGCACTGAGAAGC

pDM4-F1: CTTGCATGCGGGTAACCTGAGCTC

pDM4-R1: CTTGATATACACTCCGCTAGCGCTG

pDM4-envZ-U-F: CTAGCGGAGTGTATATCAAGCTGCGTTTCGCCGGGAA

ATCTATC

pDM4-envZ-D-R: TCAGGTTACCCGCATGCAAGTGGTGCCAATCTCCA

GCATCCCG

envZ-U-R1: CGCAAATCGGCAGAGACGCCCGCCATCAATAGCGT

envZ-U-R2: GGCGCGCAAATCGTGAGAGACGCCCGCCATCAATAGCGT

envZ-D-F1: CGTCTCTGCCGATTTGCGCACGCCGTTGACCCGT

envZ-D-F2: TCTCACGATTTGCGCGCCCCGCTGACCCGTATTCGTCTG

GCGACG

PCR primers for qRT-PCR of the *ssrA* and *ssrB* genes

Primers paired to a 101 bp region in *ssrA* gene, a 143 bp region in the *ssrB* gene, or a 109 bp region in 16*S* rRNA were used in the PCR reactions as the following:

pST16s-F1: GAGCAAGCGGACCTCATAAA

pST16s-R1: GTATTCACCGTGGCATTCTG

pSTssrA-F1: AAAACCAGTGACATTGGCTAC

pSTssrA-R1: AGCGACTCGGATCCTGCT

pSTrrsB-F1: TTAGTCTACCTGGCATCAATGG

pSTrrsB-R1: AACATAGCCATTAGCACCTGC

References

1. Anderson KM, Esadze A, Manoharan M, Bruschweiler R, Gorenstein DG, Iwahara J. Direct observation of the ion-pair dynamics at a protein-DNA interface by NMR spectroscopy. *J. Am. Chem. Soc.* **135**, 3613-3619 (2013).

2. Milton DL, O'Toole R, Horstedt P, Wolf-Watz H. Flagellin A is essential for the virulence of *Vibrio anguillarum*. *J. Bacteriol.* **178**, 1310-1319 (1996).
